# DIVORCE AND DEPRESSION: A FORENSIC CASE OF OUR OBSERVATION AND PREVENTION STRATEGIES

**DOI:** 10.1192/j.eurpsy.2023.1789

**Published:** 2023-07-19

**Authors:** C. Scalise, F. Cordasco, M. A. Sacco, V. Ritorto, G. Pulpito, S. Gualtieri, P. Ricci, I. Aquila

**Affiliations:** Institute of Legal Medicine University Magna Graecia of Catanzaro, Catanzaro, Italy

## Abstract

**Introduction:**

Nearly 300 million people worldwide are affected by depression. According to the DSM-5, the depressive episode is characterized by a depressed mood, a marked decrease in interest or pleasure in all activities, insomnia, agitation or psychomotor slowdown. It occurs mainly in the female sex. Traumatic life events are associated with a depressive onset.

**Objectives:**

It is well known that interpersonal relationships are foundations for human beings, especially emotional ones and that they have an important effect on mental health. Specifically, 60% of divorced people with a previous history of depression will develop a new depressive episode; this will develop in 10% of subjects without a previous history of depression. The recurring thought of death and suicide is also frequent, as well as the abuse of drugs and ethanol in cases of depression. The forensic pathologist often finds himself having to carry out complex inspections in order to trace the cause of death in these types of deaths.

**Methods:**

We report the case of a lady, found dead at her home, in her bed.

**Results:**

A medical prescription for benzodiazepines was found on the cabinet next to her bed with five bottles of benzodiazepines, one of which was empty, two semi-empty packs of antidepressants. Under the bed there was a photo album containing 58 pages to which the photographs of the lady’s wedding were attached. In all the photographs the groom’s face appeared torn. The hygienic-sanitary conditions of the house were precarious. The analysis of the medical record showed that the lady was being treated for major depressive disorder with psychotic manifestations, severe chronic insomnia disorder and abuse of ethyl alcohol and anxiolytics, which arose after separation from her husband five years before her. The toxicological examination performed on blood and urine confirmed the presence of massive doses of benzodiazepines and ethanol, causing death from respiratory depression.

**Conclusions:**

In similar cases, the clinical and family history as well as the toxicological examination help the forensic pathologist in defining the cause of death. Depression leads to family and social isolation, affecting all aspects of a person’s life. Divorce is not only a painful and expensive experience but also harmful to health. The subject is not only in a condition of marital and economic abandonment but also health because the resources currently used in this field are few. Together with the legal process, there should also be a health process with prevention strategies such as questionnaires, interviews, exercises in order to identify those at risk and treat them appropriately.

**Disclosure of Interest:**

None Declared

